# Renin-Angiotensin-Aldosterone System Inhibitors and COVID-19: A Meta-Analysis and Systematic Review

**DOI:** 10.7759/cureus.13124

**Published:** 2021-02-04

**Authors:** Mohab Hassib, Steven Hamilton, Ahmed Elkhouly, Yiting Li, Adam C Kaplan

**Affiliations:** 1 Internal Medicine, Saint Francis Medical Center, Trenton, USA

**Keywords:** hypertension in the global context, covid 19, cardiovascular prevention, microcirculation and inflammation

## Abstract

Introduction

Increased virulence, the severity of illness, and mortality have all been hypothesized with respect to angiotensin-converting enzyme inhibitor (ACEi)/angiotensin receptor blocker (ARB) use in coronavirus disease 2019 (COVID-19) infection. Our study aims to assess whether ACEi/ARB use in patients with COVID-19 conferred worsened severity of illness or increased mortality. Additionally, we explore the possibility of an unearthed protective benefit due to their interruption of the RAS signaling pathway as observed in cardiovascular diseases.

Methods

The Cochrane Library, MEDLINE, and EMBASE were searched for studies relevant to COVID-19 severity, mortality, and inflammation in the context of ACEi/ARB use. Eight studies were included with a total of 17,943 patients, 4,292 (23.9%) of which were taking an ACEi or an ARB. The study population was 47.9% female and the average age across all studies was 65. The studies chosen had a sample size of at least 100 patients.

Results

Mortality outcomes were assessed in six studies and showed no significant difference in mortality among the ACEi/ARB and control groups (odds ratio [OR]: 0.99, 95%CI: 0.48-2.04). Seven studies assessed the severity of COVID-19 and showed no statistically significant difference in disease severity when comparing the ACEi/ARB group to the control group (odds ratio [OR]: 1.30, 95% CI 0.87-1.94). Four studies reported the length of stay with no significant difference between the ACEi/ARB groups as compared to non-users. Four studies included inflammatory markers C-reactive protein (CRP) and D-Dimer, which were noted to be consistently lower in the ACEi/ARB groups when compared to control groups, however, this was not statistically significant.

Conclusion

Our study found no significant difference in mortality, severity of illness, or length of stay between ACEi/ARB users and non-users with COVID-19 infection. These results support the continuation of ACEi and ARBs in the setting of COVID-19 as advised by the American College of Cardiology (ACC)/American Heart Association (AHA). The decrease in CRP and D-dimer suggests a possible protective effect related to ACEi/ARB use in COVID-19, however, more studies with larger sample sizes are needed to establish this effect.

## Introduction

In December of 2019, the world was awakened to the news that a new virus, severe acute respiratory syndrome coronavirus 2 (SARS-CoV-2), had been isolated and was spreading within mainland China. By March of 2020, this virus was present worldwide and the coronavirus disease 2019 (COVID-19) pandemic had started. It was a perfect storm of globalization with a new, highly infectious coronavirus that sparked a cascade of scientific thoughts, opinions, and hypotheses. The topics ranged from prevention and treatment to potential cures of this novel disease. One of the most intriguing subjects revolved around angiotensin-converting enzyme inhibitors (ACEis) and angiotensin receptor blockers (ARBs) due to both SARS-COV-2 and ACEi/ARB’s relation to the renin-angiotensin-aldosterone signaling pathway (RAAS). The potential for increased virulence, the severity of illness, and mortality were postulated in a person who contracted SARS-CoV-2 and was being treated with an ACEi/ARB due to an upregulation of angiotensin-converting enzyme 2 (ACE2). SARS-CoV-2 uses ACE2 to gain entry to lung epithelial cells, thus promoting viral replication and intracellular transmission [1]. Furthermore, an imbalance of the RAAS to Mas signaling pathway is currently thought to promote inflammation and fibrosis due to an eventual down-regulation of ACE2 after a SARS-COV-2 infection. The combination of these mechanisms in the real world may be contributing to the acute lung injury we are observing with the current SARS-COV-2 pandemic [2]. As ACEis and ARBs are among the most common anti-hypertensive medications prescribed, up to 48% of monotherapy prescriptions [3], these concerns led to an unclear scientific message on how to manage patients currently on these medications. Many of these patients were on these medications due to their demonstrated benefits in comorbid conditions, such as heart disease, heart failure, and chronic kidney disease, for which alternative medications are not yet available. However, data from various cohorts have demonstrated conflicting results about whether ACEi/ARB use is associated with increased severity or worsened outcomes in the setting of COVID-19 infection. Our meta-analysis aims to assess whether ACEi/ARB use in patients with COVID-19 conferred worsened severity or increased mortality, and the possibility that an unearthed protective benefit exists, as hypothesized by Li et al. [4], due to a negative feedback mechanism leading to increased angiotensin 1 levels.

## Materials and methods

MEDLINE, the Cochrane Library, and EMBASE were searched up to June 9, 2020. Only articles published in English were considered for inclusion. Furthermore, abstract data were excluded and only complete observational studies that underwent the peer‐review process were included. The following Medical Subject Headings (MeSH) terms and keywords (including suffix variations of the root words) were used alone or in combination: Angiotensin-converting enzyme inhibitor(s)/ACEi, angiotensin II receptor blocker(s)/ARBs, COVID-19, SARS-CoV-2, coronavirus 2019, and 2019 novel coronavirus disease. Two investigators (Mohab Hassib and Steven Hamilton) independently assessed the identified titles for relevance. Abstracts were screened for all potentially relevant titles, and full papers were obtained for all relevant abstracts. The reference lists of the selected papers were also screened for articles that may have been missed in the initial search.

This meta-analysis was conducted and reported according to the preferred reporting items for systematic reviews and meta-analyses (PRISMA) guidelines and followed a detailed, prespecified protocol that set out using the participants, intervention, control, outcome, study design (PICOS) structure.

Studies were considered for inclusion if they met the following criteria: The study subjects were patients with both laboratory-confirmed (polymerase chain reaction) COVID-19 [1]; the intervention group included patients who were taking an ACEi or ARB prior to hospitalization, which was continued or stopped during hospitalization [2]; the control group included patients who had not taken ACEi/ARB prior to or during hospitalization [3]; the studies reported the outcomes of COVID-19 infection, including mortality, intensive care unit (ICU) admission, invasive ventilation, and length of stay [4]; the minimum number of participants was 100 in the included studies to minimize bias [5].

We collected the following information by using a standardized data extraction form: last name of the first author, publication year, study design, number of patients, patient characteristics, inflammatory markers related to COVID-19, and outcomes. The primary outcome was defined as mortality. Secondary outcomes were ICU admission, invasive ventilation, and length of stay. Data were analyzed using Review Manager Software (RevMan version 5.3; The Nordic Cochrane Center, The Cochrane Collaboration, Copenhagen, Denmark). The results were expressed in terms of odds ratio (OR) and 95% confidence intervals (95% CI).

The I2 test and associated P values were used to assess the heterogeneity of the studies. Results ranged between 0% (i.e. no observed heterogeneity) and 100%, and I2 values ≥50% were used to define a significant degree of heterogeneity. P values <0.10 according to the Cochrane Q test were considered statistically significant. All analyses were based on the random-effects model. The robustness of the meta‐analysis for publication bias was assessed by various bias indicators, including the Egger's test and the Begg's test; P values less than 0.05 indicated publication bias.

## Results

**Figure 1:**
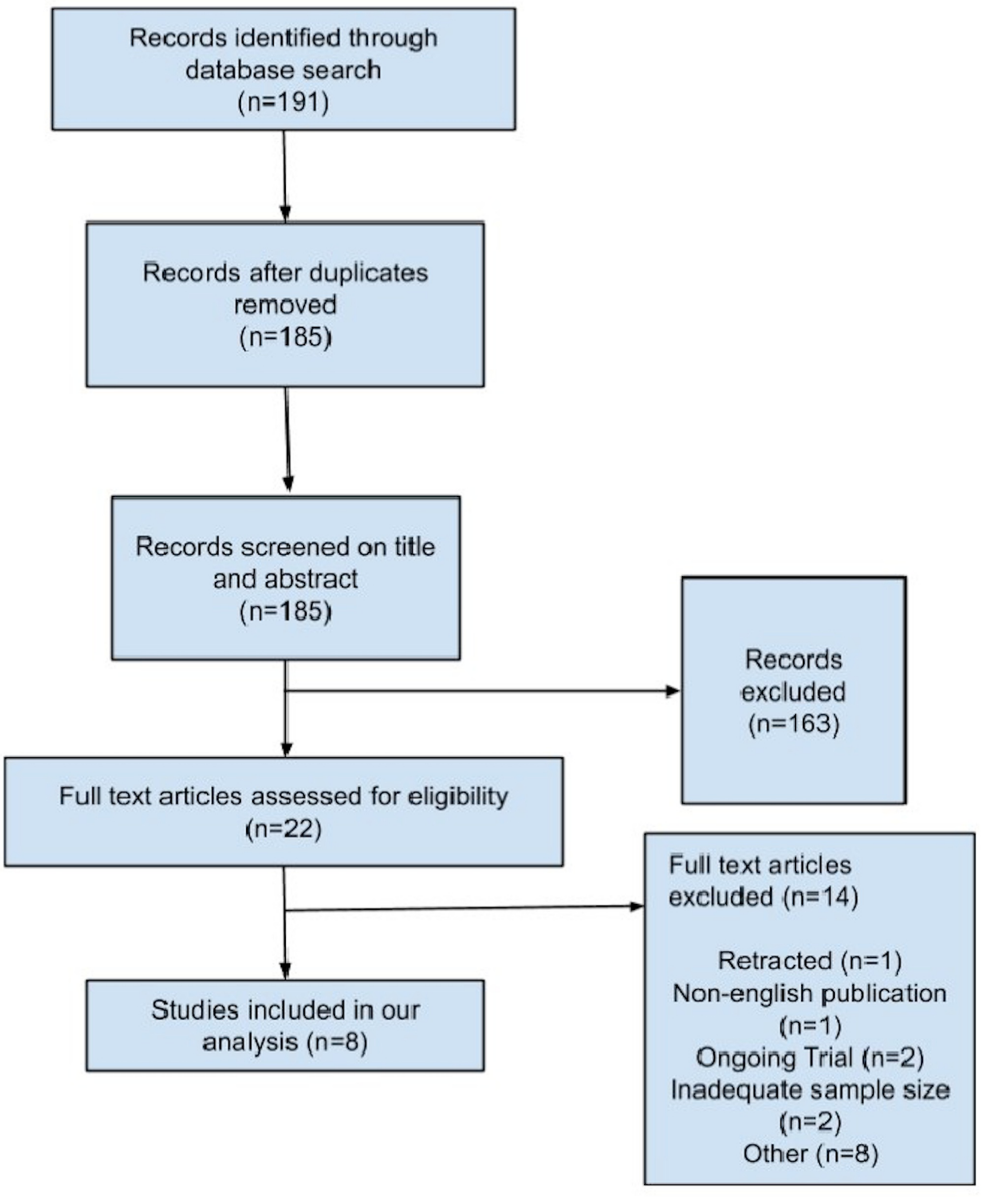
Flow chart outlining study selection

The total search included 191 studies, 169 of which were excluded after removing duplicates and reviewing the title and abstract. Subsequently, the full texts of 22 articles were reviewed. Of these, 14 articles were excluded due to non-English publication, ongoing trials, study retraction, inadequate sample size, and studies outside of the context of our analysis. A total of eight studies were included in the final meta-analysis, with a total of 17,943 patients in the study population (Figure 1). Of this study population, 4,292 (23.9%) patients were receiving an ACEi or ARB. The study population was 52.1% male and 47.9% female with an average age of 65.

All of the studies compared the mortality and clinical severity-related outcomes in COVID-19 patients on an ACEi or ARB with non-users. Unfortunately, there was no clear and uniform definition of the outcome of severity among these studies. Five studies (Tan et al. [5], Yang et al. [6], Gao et al. [7], Feng et al. [8], and Li et al. [4]) were conducted in China and defined the clinical severity of COVID-19 based on guidelines established by the National Health Commission of the People’s Republic of China (7th edition). In the majority of the studies, age and sex were matched in both the control and ACEi/ARB groups. Comorbidities, including coronary artery disease, hypertension, diabetes mellitus, and chronic kidney disease, were fairly distributed among both ACEi/ARB and control groups; such an observation helped minimize any confounding that may affect severity or mortality (Table 1).

**Table 1:** Demographics and clinical characteristics of patients in our included studies Retro=Retrospective Study; CS=Cohort Study; CCS=Case Control Study; COVID-19=coronavirus disease 2019; ACEi=Angiotensin-Converting Enzyme Inhibitor; ARB: Angiotensin Receptor Blocker; COPD=Chronic Obstructive Pulmonary Disease; CKD=Chronic Kidney Disease *Mean **Mean age among all COVID-19 positive patients

Study	Study design	Country	Female (%)	Patient age (median)(n)		Total sample size (n)	Patients taking ACEi/ARB (n)	Control Group (n)	Patient Co-morbidities (%)		Primary indication for ACEi/ARB use	Treatment received			
				ACEI/ARB group	Control group				ACEI/ARB group	Control group		Antiviral (%)	Hydroxychloroquine (%)	Antibiotics (%)	Steroids (%)
Tan et. al 2020 (5)	Retro	CHN	49	67	67.5	100	31	69	Diabetes Mellitus 25.8, Chronic renal disease 12.9, Coronary artery disease 16.1, COPD 6.5	Diabetes Mellitus 29, Chronic renal disease 7.2, Coronary artery disease 18.8, COPD 10.1	Hypertension	ACE group(19), non-ACE group(52)	ACE group (0), non-ACE group (0)	ACE group (17), non-ACE group (40)	ACE group (3), non-ACE group (26)
Yang et. al 2020 (6)	Retro	CHN	50.8	65	67	126	43	83	Diabetes mellitus 30.2, Chronic renal disease 0, Cardiovascular disease 16.3, Respiratory disease 7, hepatic disease 7, Neurological disease 9.3	Diabetes mellitus 30.1, Chronic renal disease 3.6, Cardiovascular disease 19.3, Respiratory disease 3.6, Hepatic disease 6.1, Neurological disease 7.2	Hypertension	ACE group (30), non ACE group(66)	ACE group (0), non-ACE group (0)	ACE group (29), non-ACE group (69)	ACE group (16), non-ACE group (23)
Mehta et. al 2020 (10)	CS	USA	45	64*	53*	1735	212	1523	Diabetes mellitus 52, Coronary artery disease 22, Heart failure 16, Hypertension 94, Respiratory disease 14, Obesity 50	Diabetes Mellitus 36, Coronary artery disease 18, Heart failure 17, Hypertension 76, Respiratory disease 13, Obesity 53	Hypertension, Heart failure, Diabetes mellitus, Coronary artery disease	N/A	N/A	N/A	N/A
Mancia et al. 2020 (11)	CCS	ITA	36.7	68**		6272	2896	3376	N/A	N/A	Hypertension, Heart failure, Diabetes mellitus, Coronary artery disease	N/A	N/A	N/A	N/A
Feng et. al 2020 (8)	Retro	CHN	43.1	53***		476	33	443	Diabetes mellitus 10.3, Cardiovascular disease 8, Malignancy 2.5, Hypertension 29.2, Respiratory disease 4.6, Cerebrovascular disease 3.6, Co-morbidities for all COVID-positive patients		Hypertension	60.1	0	67	26.7
Gao et. al 2020 (7)	Retro	CHN	48.9	62*	64*	2877	200	2677	Hypertension 100, Diabetes Mellitus 30.1, Angina 17.5, Myocardial Infarction 0, Heart failure 0.5, COPD 0.6, Stroke 3.3, Renal failure 1.1	Hypertension 100, Diabetes Mellitus 26.6, Angina 15.2, Myocardial infarction 0.6, Heart failure 1.5, COPD 1.5, Stroke 3.8, Renal failure 1.9	Hypertension	N/A	N/A	N/A	N/A
Jung et. al 2020 (9)	CS	KOR	56	62.5*	41.5*	5179	762	4417	Hypertension 94, Diabetes mellitus 48, Myocardial Infarction 4, Heart failure 14, Chronic lung disease 40, Cerebrovascular disease 19, CKD 19	Hypertension 10, Diabetes mellitus 11, Myocardial Infarction 1, Heart failure 3, Chronic lung disease 27, Cerebrovascular disease 4, CKD 3	Hypertension, Diabetes mellitus, Chronic kidney disease	40	21	31	4
Li et. al 2020 (4)	Retro	CHN	53.7	65	67	1178	115	1063	Hypertension 100, Diabetes mellitus 36.5, Coronary artery disease 23.5, Heart failure 4.3, Lung disease 7, Cerebrovascular disease 23.5, Neurological disease 2.6, CKD 11.3	Hypertension 100, Diabetes mellitus 34.4, Coronary artery disease 14.2, Heart failure 2, Lung disease 4, Cerebrovascular disease 16.6, Neurological disease 10.1, CKD 8.9	Hypertension				

**Figure 2:**
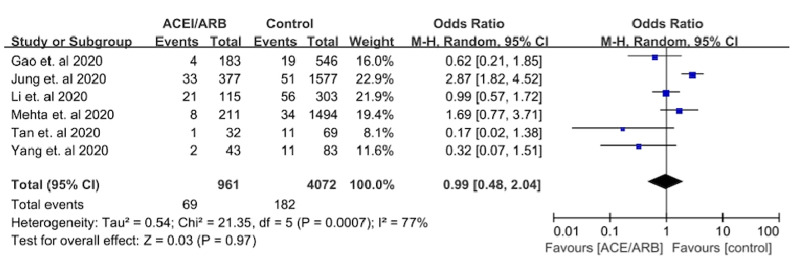
Forest plot depicting the meta-analysis of mortality outcomes in COVID-19 patients on an angiotensin-converting enzyme inhibitor (ACEi)/angiotensin receptor blocker (ARB) COVID-19=coronavirus disease 2019

**Figure 3:**
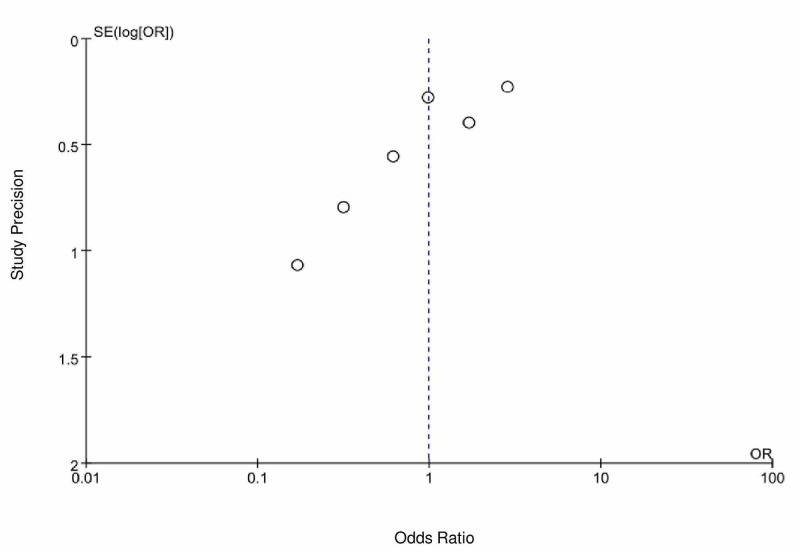
Funnel plot depicting publication bias for studies evaluating mortality outcomes in COVID-19 patients on an angiotensin-converting enzyme inhibitor (ACEI)/angiotensin receptor blocker (ARB) COVID-19=coronavirus disease 2019

Mortality outcomes were assessed in six studies: Gao et al. [7], Jung et al. [9], Li et al. [4], Mehta et al. [10], Tan et al. [5], and Yang et al. [6]. In Gao et al. [7], Tan et al. [5], and Yang et al. [6], a lower mortality event rate was observed in the ACEi/ARB group as compared to the control group, with an odds ratio of 0.62 [95% CI 0.21-1.85], 0.17 [ 95% CI 0.02-1.38], and 0.32 [95% CI 0.07-1.51], respectively. In contrast, Jung et al. [9] and Mehta et al. [10] showed a higher mortality event rate in the ACEi/ARB group as compared to the control group with an odds ratio of 2.87 [95% CI 1.82-4.52] and 1.69 [0.77-3.71], respectively. In a pooled analysis of six peer-reviewed studies, there was no statistical significance of mortality between the ACEi/ARB group as compared to the control group with an OR of 0.99 (95% CI = 0.48-2.04, I2 = 77, P-value = 0.97) (Figure 2). There is possible minimal publication bias seen in Figure 3.

**Figure 4:**
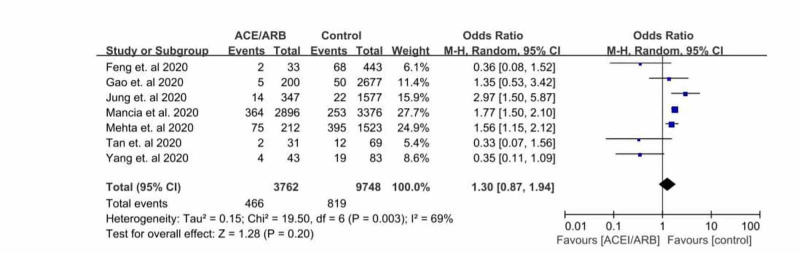
Forest plot depicting the meta-analysis of clinical severity in COVID-19 patients on an angiotensin-converting enzyme inhibitor (ACEi)/angiotensin receptor blocker (ARB) COVID-19=coronavirus disease 2019

**Figure 5:**
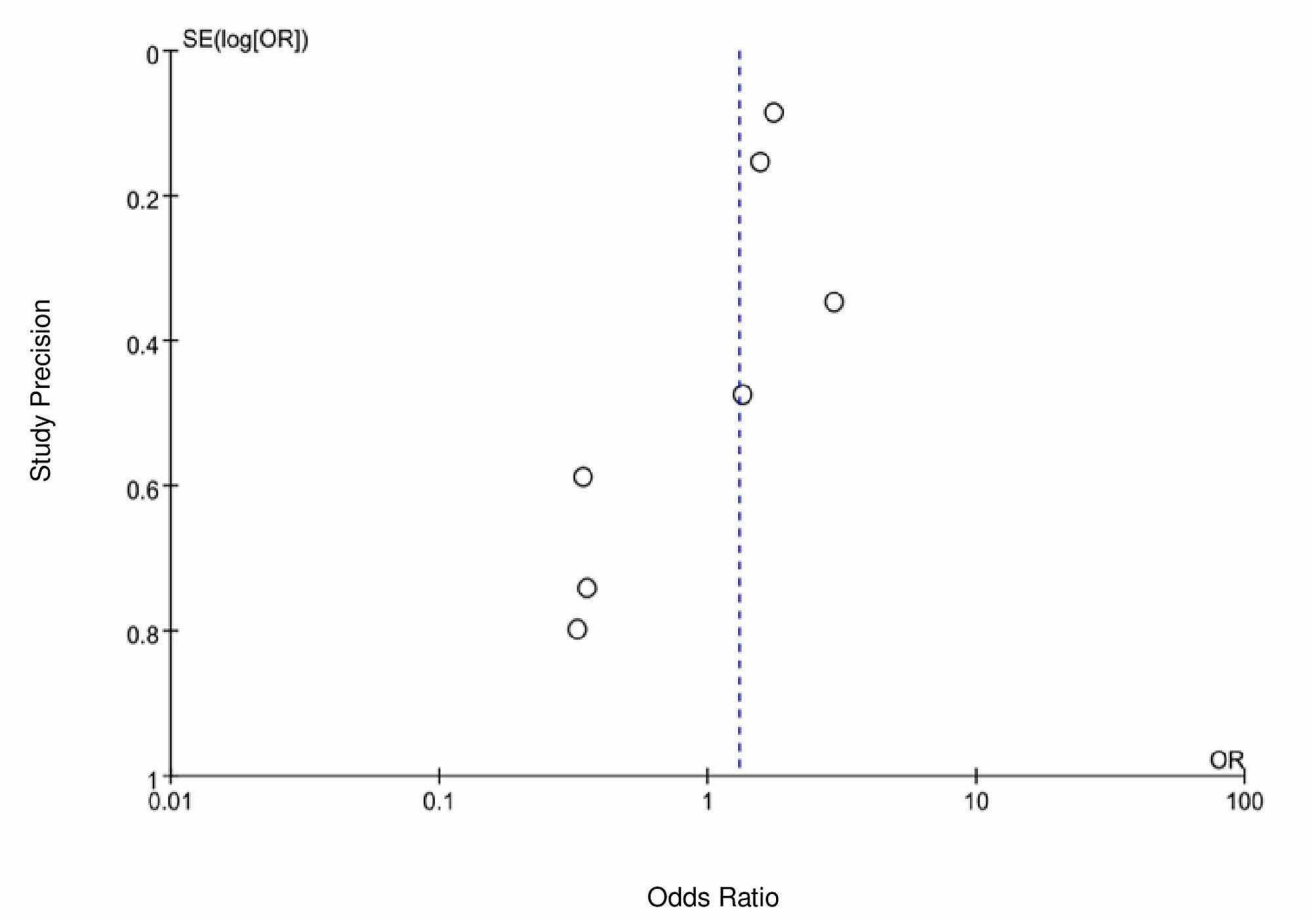
Funnel plot depicting publication bias for studies evaluating clinical severity based on Chinese guidelines in COVID-19 patients on an angiotensin-converting enzyme inhibitor (ACEi)/angiotensin receptor blocker (ARB) COVID-19=coronavirus disease 2019

Association of the severity of COVID-19 between ACEi/ARB and the control group was assessed in seven studies: Feng et al. [8], Gao et al. [7], Jung et al. [9], Mancia et al. [11], Mehta et al. [10], Tan et al. [5], and Yang et al. [6]. There was no clear and uniform definition for severity outcome in these studies. In our study, we defined severe COVID-19 infection as ICU admission, use of mechanical ventilation, or septic shock. In a pooled analysis of seven peer-reviewed studies, the use of an ACEi/ARB showed no statistically significant association in disease severity versus non-users (OR = 1.30, 95% CI 0.87-1.94 I2 = 69 and P-value = 0.20), which is portrayed in Figure 4. There is a possible minimal publication bias seen in Figure 5.

Four studies reported the length of stay. Tan et al. [5] and Li et al. [4] reported the length of stay as a median while Yang et al. [6] and Feng et al. [8] reported it as a mean. There was no difference in overall length of stay between the ACEi/ARB groups as compared to non-users, which can be seen in Table 2. The exception to this was the study by Feng et al. [8], which showed a lower overall length of stay in comparison to Tan et al. [5] and Yang et al. [6].

**Table 2:** Comparison of overall length stay in COVID-19 patients on an angiotensin-converting enzyme inhibitor (ACEi) and/or angiotensin receptor blocker (ARB) vs. non-users *Median COVID-19=coronavirus disease 2019

Study	ACEi/ARB group	Non-ACEI/ARB group	Overall length of stay
Tan et. al 2020* (5)	33	36.5	N/A
Yang et. al 2020 (6)	35.2±12.8	37.5±12.3	36.7 +/-12.4
Feng et. al 2020 (8)	N/A	N/A	16 (12-24)
Li et. al 2020* (4)	19	N/A	N/A

There was no significant difference in the levels of C-reactive protein (CRP) and D-dimer in the ACEi/ARB groups as compared to control groups, as seen in Table 3. Although Yang et al. [6] did show a lower CRP level in ACEi/ARB as compared to control with a P-value of < 0.49, other studies failed to show similar findings.

**Table 3:** Comparison of inflammatory markers COVID-19 patients on an angiotensin-converting enzyme inhibitor (ACEi) and/or angiotensin receptor blocker (ARB) vs. non-users. *Median CRP=C-Reactive Protein; COVID-19=coronavirus disease 2019

Study	CRP		D-dimer	
	ACEI/ARB Group	Control Group	ACEI/ARB Group	Control Group
Tan et. al 2020* (5)	23.97 (4-43) (n=31)	24 (6.7-62.5) (n=69)	0.76 (0.26-2.39) (n=31)	0.96 (0.58-0.21) (n=31)
Yang et. al 2020* (6)	11.5 (4.0–58.2) (n=43)	33.9 (5.1–119.2) (n=83)	0.40 (0.30–0.61) (n=43)	0.47 (0.29–1.82) (n=31)
Gao et. al 2020 (7)	2.72 (1.18-11.67)	3.18 (1.07-12.29)	0.45 (0.27-0.94)	0.53. (0.27- 1.06)
Li et. al 2020 (4)	2.1 (0.3 - 5.2)	2.6 (0.4 - 6.0)	0.7 (0.4 - 1.6)	1.7 (0.3 - 2.5)

## Discussion

Despite the physiological basis that underlies concerns for the use of ACEi/ARBs in the context of COVID-19 infection, our study found no significant difference in mortality, severity, or length of stay between patients with ACEi/ARB exposure and those without.

We included eight studies with a minimum sample size of 100 participants, which allowed us to analyze 17943 patients. Our findings, while having moderate to high heterogeneity, have been in line with other recently published data, which also did not reveal a signal for harm associated with exposure to ACEi/ARBs in COVID-19 infection [12-13]. These results further support the AHA, ACC, Centers for Disease Control and Prevention (CDC), and World Health Organization position statements that ACEi/ARBs should be continued in COVID-19 infection in patients already taking them at the time of diagnosis unless another indication for discontinuation is present, other than SARS-COV-2 positivity. 

Much of the concern involving SARS-COV-2 and ACEi/ARBs revolves around RAAS signaling imbalance causing inflammation due to ATR1 activation. Studies performed by Hayiroglu et al. [14], Gormez et al. [15], and Al-Samkari et al. [16] have revealed a linear relationship with inflammatory markers and severity of illness. Therefore, potential treatment targets for this virus may revolve around limiting RAAS activation. This led researchers, including us, to look into the available data to see if there is an association with ACEi/ARB use and a possibility of lower levels of inflammation due to their interactions within the RAAS signaling pathway. Our results, which consisted of data from four of the eight studies, showed a consistent decrease in both D-dimer and CRP levels in ACEi/ARB-exposed patients as compared to controls. These differences were not statistically significant but if the linear relationship between inflammation and severity of illness is true then our study would support the notion that ACEi/ARB use for RAAS blockade may be beneficial in patients with COVID-19 infection. Zhang et al.’s study [17], which studied patients taking ACEi/ARBs for hypertension, a significant decrease in severity and mortality was observed, which supports our conclusion.

There was no difference in overall length of stay between the ACEi/ARB groups compared to the control groups, with the exception of the study by Feng et al. [8], which showed a lower overall length of stay in comparison to Tan et al. [5] and Yang et al. [6]. This may be due to smaller sample size or a younger patient population with a mean age of 53 in Feng et al.’s study [8]. Further studies are therefore required to demonstrate any possibility of a difference in length of stay with ACEi/ARB users with COVID-19 infection.

There are a number of potential limitations of our study. One, there was no differentiation between ACEi and ARBs with regards to the outcomes measured; the studies we analyzed grouped the use of ACEi and ARBs as a single entity. Though these medications are usually grouped together and used synonymously in practice, they have different modes of action and if studied individually in the context of COVID-19 could yield different outcomes, which is potentially an area for further research. Currently, there is conflicting data on this topic, as Flacco et al’s study [18] suggested that results did not differ when ACEis and ARBs were analyzed separately versus together in this context, whereas Pranata et al.’s study [19] specifically found that the ARB group showed decreased mortality but not the ACEi group. A second limitation is the heterogeneity of the definition of severe COVID-19 infection among the studies analyzed. For example, Mehta et al. [10] described severe infection as ICU admission or use of mechanical ventilation, whereas Mancia et al. described severe disease as ICU admission or death [11]. This discordance is expected among studies, which led us to construct our own definition of severe COVID-19 infection to be used as an outcome. The aim was to accurately represent severe COVID-19 infection across all studies chosen for our analysis, however, some patients from our chosen studies will have not met our study's criteria for severe infection. Lastly, while we showed a decrease in inflammatory markers in our ACEi/ARB-exposed group, this finding likely relied on an included study by Yang et al. [6], and, therefore, the true correlation of our findings to clinical practice should be taken more as a means to guide further research rather than change current practice.

## Conclusions

Our study found no significant difference in mortality, severity, or length of stay between ACEi/ARB users and non-users with COVID-19 infection and therefore supports the continuation of ACEis and ARBs in the setting of COVID-19. The decrease in CRP and D-dimer may suggest a protective effect related to ACEi/ARB use in COVID-19; however, more studies with larger sample sizes are needed to establish this effect.
